# Proceedings and Discussions of the Cincinnati Dental Association

**Published:** 1860-05

**Authors:** 


### Proceedings of Societies.

### PROCEEDINGS AND DISCUSSIONS OF THE CINCINNATI DENTAL ASSOCIATION.

THE regular monthly meeting of this association was held at the office of Drs. Wardel & Doughty, March 18th, 1860. President Wardle in the chair.

Minutes of the last meeting were read and approved.

The name of Dr. J. A. Watling was presented for membership. Upon balloting, he was found unanimously elected to membership.

This being the regular evening for the election of officers, proceeded to the election, which resulted as follows:

Dr. J. TAFT, *President.*

Dr. H. A. SMITH, *Recording Secretary.*

Dr. J. RICHARDSON, *Corresponding Secretary.*

Dr. WARDLE, *Treasurer.*

Drs. RICHARDSON, H. A. SMITH, J. G. CAMERON, *Publication Com.*

Reports were called for, but the committees not being prepared to report, proceeded to other business.

DISCUSSIONS.

The subject: *Hemorrhage after the extraction of teeth. Its conditions, causes and remedy.*

Dr. FOOTE opened the discussions, by saying that the proximate cause is an irruption of blood vessels. The remote cause is a strumous or ill condition of the system, which does not possess sufficient recuperative power.

The remedy most generally effective, is the application of pressure, but this will not always effect the desired purpose; he found very minute doses of quinine to be useful, dissolved in water and taken internally.

Dr. RICHARDSON has only met with two or three difficult cases, one probably caused by an interruption of the menstrual flow, the other by a fractured and lacerated condition of the tissues, thus preventing a contraction of the parts. He referred to perchloride of iron, as being a good hemostatic. It may be introduced with a plug of cotton or linen. Failing in this, by this method, he would take an impression of the parts, and form a plate, and use it as an instrument of compression, using it in connection with pledgets and roll. In cases of predisposition to hemorrhage, it might be necessary to resort to some constitutional treatment, perhaps give some astringents internally. Persons of plethoric habit might require purgatives.

Dr. DAVENPORT had experienced some difficulty with one case, where an upper wisdom tooth had been extracted, but succeeded in arresting the flow chiefly by compression.

Dr. WARDLE had met with three difficult cases, but was successful in arresting the hemorrhage by local treatment; used the perchloride of iron.

Dr. SMITH gave an analysis of the blood. In the blood we have several component principles, prominent among which are albumen and fibrine. He explained the progress of coagulation; it does not occur so readily after prolonged bleeding. Iron enters largely into the blood. Clot acquires firmness in proportion to the amount of fibrine it contains; no coagulation takes place in persons killed by lightning. Blood last drawn coagulates more rapidly, but not so firmly, as that first drawn. Fibrine is below the standard during pregnancy, especially from the first to the fourth month. A decrease of fibrine increases the tendency to hemorrhage. A hemorrhagic predisposition may be traced to the state of the blood; it may be active or passive.

*Treatment.*—Cold water is effectual, inasmuch as it produces contraction of the capillaries. Most styptics are astringents, but some astringents are not styptics. Nitrate of silver is a powerful astringent.

Dr. TAFT remarked that hemorrhage depends upon one or more of several conditions. In a hemorrhagic diathesis, there is a want of tone, or contractile power in the coats of the vessels, and hence in a rupture or laceration, there is no contraction of the wounded vessel, owing to inability. The blood is often-times changed to such an extent, that it does not coagulate regularly or firmly on exposure to the atmosphere. Either of these conditions will be operative in the production of hemorrhage from any ruptured vessels. Hemorrhage sometimes occurs where there is no obvious hemorrhagic diathesis, in consequence of the rupture of some considerable vessel. The coats of a vessel are sometimes so firmly attached to the walls of a bony canal through which it passes, that it is impossible for it to contract as in soft parts. When an arrangement of this kind exists in connection with a vitiated condition of the blood, it constitutes a difficult case. There are many conditions that will modify the tendency to hemorrhage, such as various diseased conditions, residence in malarious districts, or where there is anything that will vitiate the vitality and disease the blood. He remarked that the greatest number of cases of hemorrhage with which he has had to contend were from malarious regions; did not remember that he had ever witnessed a case of obstinate hemorrhage from the high and healthy parts of the country. This is a particular that should not be lost sight of, by those who practice in portions of the country where miasma exists. The treatment of hemorrhage is either general or local, or both, as the indications may direct. Constitutional treatment should be such as would give tonicity and contractile power to the vascular system, and improve the blood so as to facilitate its coagulability. The local treatment consists in the application of astringents, styptics and the compress, the application of which is pretty well understood.

Dr. Foote would not expect hemorrhage from extraction of a tooth, unless there was a scorbutic or flabby condition of the system, or some mechanical arrangement that would facilitate the hemorrhage. Spoke of the condition of the blood of those having what is called Green Sickness. It is a want of iron in the blood. He also suggested the use of electricity in cases of hemorrhage, giving some directions how to apply it.

Did not favor the use of acetate of lead, but preferred the preparations of iron—persulphate, perchloride of iron, etc.

Dr. Richardson mentioned nitric acid as a valuable agent in many cases. He referred to the various modes of treatment at some length.

Some remarks were made by some of the members in regard to the use of the mallet in filling teeth. They were, however, characterized by a spirit of inquiry.

Adjourned to meet on the second Tuesday of April, at the office of Drs. Taft & Foote. R. H.

